# Wash-resistance of pirimiphos-methyl insecticide treatments of window screens and eave baffles for killing indoor-feeding malaria vector mosquitoes: an experimental hut trial, South East of Zambia

**DOI:** 10.1186/s12936-018-2309-2

**Published:** 2018-04-13

**Authors:** Dingani Chinula, Chadwick H. Sikaala, Pascalina Chanda-Kapata, Busiku Hamainza, Reuben Zulu, Lisa Reimer, Elizabeth Chizema, Samson Kiware, Fredros O. Okumu, Gerry Killeen

**Affiliations:** 1 National Malaria Elimination Centre, Chainama Hills Hospital Grounds, PO Box 32509, Lusaka, Zambia; 2 Ministry of Health, Directorate of Public Health, Lusaka, Zambia; 3 Vector Biology Department, Liverpool School of Tropical Medicine, Pembroke Place, Liverpool, L35QA UK; 4 Environmental Health and Ecological Sciences Department, Ifakara Health Institute, Kiko Avenue, PO Box 53, Ifakara, United Republic of Tanzania; 5 School of Public Health, University of the Witwatersrand, Parktown, Johannesburg, South Africa; 6 Institute of Biodiversity, Animal Health and Comparative Medicine, University of Glasgow, Glasgow, G12 8QQ UK

**Keywords:** Vector control, Indoor-feeding, Mosquito, *Anopheles*, Malaria, *Plasmodium*, Insecticide, Resistance, Houses

## Abstract

**Background:**

The effectiveness of long-lasting insecticidal-treated nets (LLINs) and indoor residual spraying (IRS) for malaria control is threatened by resistance to commonly used pyrethroid insecticides. Rotations, mosaics, combinations, or mixtures of insecticides from different complementary classes are recommended by the World Health Organization (WHO) for mitigating against resistance, but many of the alternatives to pyrethroids are prohibitively expensive to apply in large national IRS campaigns. Recent evaluations of window screens and eave baffles (WSEBs) treated with pirimiphos-methyl (PM), to selectively target insecticides inside houses, demonstrated malaria vector mortality rates equivalent or superior to IRS. However, the durability of efficacy when co-applied with polyacrylate-binding agents (BA) remains to be established. This study evaluated whether WSEBs, co-treated with PM and BA have comparable wash resistance to LLINs and might therefore remain insecticidal for years rather than months.

**Methods:**

WHO-recommended wire ball assays of insecticidal efficacy were applied to polyester netting treated with or without BA plus 1 or 2 g/sq m PM. They were then tested for insecticidal efficacy using fully susceptible insectary-reared *Anopheles gambiae* mosquitoes, following 0, 5, 10, 15, then 20 washes as per WHO-recommended protocols for accelerated ageing of LLINs. This was followed by a small-scale field trial in experimental huts to measure malaria vector mortality achieved by polyester netting WSEBs treated with BA and 2 g/sq m PM after 0, 10 and then 20 standardized washes, alongside recently applied IRS using PM.

**Results:**

Co-treatment with BA and either dosage of PM remained insecticidal over 20 washes in the laboratory. In experimental huts, WSEBs treated with PM plus BA consistently killed similar proportions of *Anopheles arabiensis* mosquitoes to PM-IRS (both consistently ≥ 94%), even after 20 washes.

**Conclusion:**

Co-treating WSEBs with both PM and BA results in wash-resistant insecticidal activity comparable with LLINs. Insecticide treatments for WSEBs may potentially last for years rather than months, therefore, reducing insecticide consumption by an order of magnitude relative to IRS. However, durability of WSEBs will still have to be assessed in real houses under representative field conditions of exposure to wear and tear, sunlight and rain.

**Electronic supplementary material:**

The online version of this article (10.1186/s12936-018-2309-2) contains supplementary material, which is available to authorized users.

## Background

The main malaria vector control strategies used today are long-lasting insecticidal nets (LLINs) and indoor residual spraying (IRS), which together account for most of the 1.3 billion fewer malaria cases and 6.8 million fewer malaria-related deaths that occurred due to declining transmission between 2000 and 2015 [1–3]. However, the effectiveness of IRS and LLINs are threatened by insecticide resistance against the four insecticide classes (carbamates, pyrethroids, organo-chlorines, organophosphates) that already have full recommendations from the World Health Organization (WHO) [4–7]. Excessive reliance upon pyrethroids, one of the most affordable options, has selected for resistance to this active ingredient among *Anopheles* mosquito populations, thus compromising vector control across most parts of Africa [6, 7]. The current global plan for insecticide resistance management (GPIRM) recommends the use of mixtures of insecticides with complimentary mode of actions for LLINs [4] some of which have recently been developed [8]. In the meantime, IRS remains the only widely accepted format for delivering rotations, mosaics or combinations (when combined with LLINs) [4]. Many countries have developed insecticide resistance management plans aligned with the GPIRM, but few of them have implemented such approaches in practice, mostly because they are too expensive to implement across national scales [9–11].

The only insecticide available for IRS in Zambia at the time of this study, to which all malaria vector populations surveyed remained susceptible, was the organophosphate pirimiphos-methyl (PM) [12–14]. One 833-ml bottle of the preferred micro-encapsulated formulation of PM (Actellic 300CS) covers approximately 250 sq m of indoor wall and ceiling surfaces at the recommended rate of 1 g/sq m, but costs approximately $23.34, exclusive of shipping and importation costs (Zambian National Malaria Elimination Centre (NMEC), pers. comms). It is therefore prohibitively expensive for most malaria-endemic countries to apply as IRS at nationwide universal coverage targets. For example, even in the sparsely populated southern African country of Zambia, spraying the 3,281,046 million eligible structures [15] would require 911,818 bottles per spray round per year. Inclusive of international shipping costs $1.16 per bottle (NMEC pers. comms) and public-sector procurement subsidies, supplying the country with PM would cost $22.3 million, even before accounting for in-country transportation, equipment, disposal, and labour. The carbamate, bendiocarb, the other major alternative to pyrethroids and organo-chlorines lasts for only 2–3 months and requires frequent re-applications, therefore making it similarly expensive [1]. The growing resistance-driven need for such costly insecticides has resulted in IRS coverage being scaled down globally from 5.7% in 2010 to 3.1% in 2015 [1]. The extent of this IRS coverage contraction has been most notable in sub-Saharan Africa from 10.5 to 5.7% [1, 10, 11] and this has caused a rebound of malaria burden in some settings [16].

**Fig. 1:**
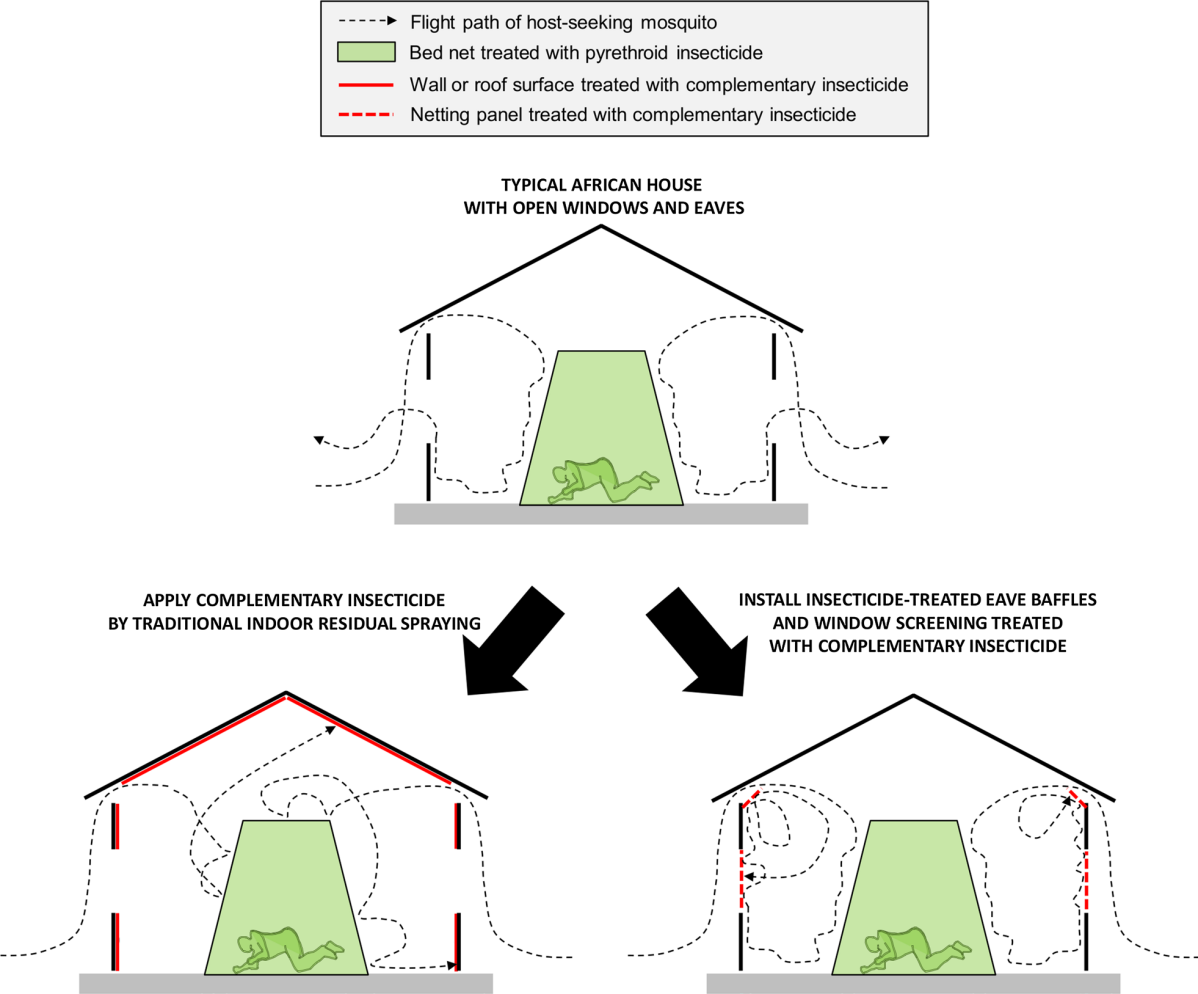
A schematic illustration of the mechanisms of action of traditional indoor residual spraying, as well as window screens and eave baffles, as methods for delivering complementary non-pyrethroid insecticides to houses with open eaves and windows

To overcome these challenges, the same insecticide formulations for IRS have recently been more selectively applied to netting window screens and eave baffles (WSEBs) installed in experimental huts (Fig. 1) [17]. Netting window screens are familiar to most residents of tropical countries, which prevent mosquitoes from entering houses, but also block their exiting once they have found their way inside. However, eave baffles had previously only been used almost exclusively as a methodological tool in experimental hut studies of mosquitoes. Eave baffles consist of netting panels that slant upwards and inwards from top of the wall towards the roof, but leaving a small gap that mosquitoes readily find when they are entering huts but not when they try to exit [18]. Targeting these entry and exit points for mosquitoes required far lower quantities of insecticides than IRS application to all internal wall and ceiling surfaces of the same standardized huts [15].

WSEBs therefore have potential to be used as an alternative to IRS, with the potential advantage of allowing affordable use of insecticide mixtures for insecticide resistance management. In the context of rural Tanzanian houses, WSEBs require treatment of only one-fifth the surface area required by IRS [17], and may be co-treated with polyacrylate binding agents, so that the durability of insecticide treatments could potentially be extended. By reducing insecticide consumption rates in these two complementary ways, WSEBs could potentially enable affordably simultaneous deployment of multiple active ingredients rotations, mosaics, combinations, or even mixtures [17]. However, previous experiments in Tanzania were merely intended to establish proof-of-concept for WSEBs, demonstrating that they are efficacious in controlling *Anopheles* malaria vectors. Indeed, the preceding study in Tanzania was not extended for long enough to assess the longevity of insecticide on netting material, and the quantities of BA used may have been too low for this insecticide.

The overall aim of this evaluation was therefore to demonstrate that WSEBs, co-treated with PM and BA doses known to retain this particular insecticide formulation on netting, can remain efficacious for killing malaria mosquitoes even after multiple washes, similar to those recommended by WHO for evaluating LLIN durability [19]. While it is not expected that WSEBs would be repeatedly removed and washed following installation in normal houses under programmatically relevant field conditions of normal use, such repeated washing is the only approach to accelerated ageing of insecticides on netting materials for evaluating durability of efficacy that is recommended by the WHO [19].

## Methods

### Laboratory assays

WHO-standardized wire ball assays [19] were conducted to optimize the dosage of binder used for extending the durability of PM treatments. Tests were conducted in the entomology laboratory at the National Malaria Elimination Centre situated in Lusaka, Zambia. Netting panels of 0.135 sq m, made from 100-denier polyester multifilament mesh with 156 holes/sq in, were treated with either 0 (negative control), 1, or 2 g/sq m micro-encapsulated PM (Actellic 300CS^®^, Syngenta AG) plus 17.3 ml/sq m of the binding agent from the same company. Additional experiments included 1 or 2 g/sq m micro-encapsulated PM without the binding agent. The binding agent is usually provided as part of a lambda-cyhalothrin based product, called Icon Maxx^®^ (Syngenta AG), used to extend the durability of long-lasting treatment of bed nets [20]. This dosage of BA was 14 times higher than that recommended for co-treatment with lambda-cyhalothrin, and was calculated based on the example for micro-encapsulated PM described in the patent for this technology [21]. The treatments were washed for 5, 10, 15, and then 20 times in 500 ml of 2 g/l of a locally available detergent commonly used for washing clothes and bed nets (Boom^®^, Trade Kings), which was completely suspended in distilled water in an aluminium vessel and agitated with a laboratory shaker. However, it is important to note that the WHO protocol was adapted to local conditions, as a locally available detergent was used instead of Savon de Marseille. The netting pieces were washed and rinsed twice for 10 min in 500 ml of distilled water. After washing, the nets were hung and allowed to dry in a dark room for 12 h and then stored in aluminium foil at room temperature. For each of the two PM treatments and each negative control, two replicate batches of 11 female 2–5 days old *Anopheles gambiae* from a fully pyrethroid-susceptible Kisumu strain colony, were exposed for 3 min to wire balls, which the treated panels were wrapped around. Total mortality rates after 24 h were recorded according to standard guidelines [19].

### Field experiments

The experiments were conducted in Chisobe village in Luangwa District, Southeast of Lusaka in Zambia (15.6265°S, 30.4041°E). IRS with PM at 1 g/sq m had been applied to all eligible structures between December 2016 and January 2017, immediately prior to this study. Historically, the main vector responsible for malaria transmission in this setting has been indoor-feeding *Anopheles funestus*, which are highly resistant to pyrethroids, and to a lesser extent carbamates, but not organophosphates or organochlorines [13, 22, 23]. A number of other species from within the *An. funestus* group, the *An. gambiae* complex, and several other taxa also mediate much lower transmission levels [24].

This small-scale field trial was conducted in four experimental huts of the Ifakara design [25, 26]. A total of eight adult male volunteers were recruited to sleep in the huts overnight from 19.00 to 07.00. Each pair of sleepers was assigned to a single specific hut for the duration of the experiment. This was done to combine hut and human participant effects into a single measurable source of variation that can be accounted for with a single random effect in generalised linear mixed models (GLMM), requiring only a single degree of freedom to maximize statistical power. Each of the eight participants slept under an intact LLIN (PermaNet 2.0^®^, Vestergaard Frandsen).

Two types of WSEBs were used [17]: (1) negative control, untreated sets with window screens and eight eave baffle pieces, with a combined total surface area of about 6 sq m, that allowed mosquitoes to enter through half of the eave gaps but allowed them to freely exit the house and be captured in exit traps placed over the windows and the other unscreened half of the eave gaps; or, (2) PM-treated sets with screens over all the windows and 16 eave-baffle pieces covering all the eave gaps, with a combined total surface area of about 12 sq m, to allow mosquitoes to enter through the eaves but then completely block their exit via windows or eaves with netting panels that were treated with PM and BA. These 8-piece and 16-piece WSEB types were used for the untreated negative controls and for insecticide-impregnated test treatments, respectively.

The treatment arrangements for each round of replication of the study design are summarized in Table 1. Two replicate sets of 16-piece WSEBs were co-treated with 2 g/sq m PM and 17.3 ml/sq m of BA, as described above for the wire ball assays, and labelled as T1 and T2. The PM dosage selected was twice that recommended for IRS, and was based on the slightly superior results obtained from previous experimental hut trials of WSEBs [17], rather than the results of the wire ball assays described above, which suggested no advantage of this higher dosage. Four replicates of 8-piece untreated WSEBs labelled as U1, U2, U3, and U4 acted as negative controls. Hut A and C were randomly selected to be sprayed with 1 g/sq m of PM according to standard IRS guidelines and fitted with 8-piece sets of untreated WSEBs (U1 and U2), which were then exchanged between these two IRS-treated huts every day for the duration of the study. Hut B and D were only sprayed with water (negative control for IRS) and each was fitted with either a 16-piece WSEB set treated with PM plus BA or an 8-piece untreated WSEB set, and these two alternative treatments were exchanged every day. The replicates of each treatment (T1 for T2 and vice versa, U3 for U4 and vice versa) were exchanged every 2nd day, staggered by 1 day to days 2 and 4 of the replication cycle, so that exchange of treatment and replicate were not covariant. This treatment rotation schedule for WSEBs in these IRS-free huts therefore consisted U3 and T1 on rotation replicate day 1 and 4, but U4 and T2 on days 2 and 3 (Table 1). One full replicate of rotation through these four arrangements was accomplished over 4 nights, resulting in each of the 4 WSEB treatment replicates relevant to water-sprayed huts (T1, T2, U3, U4) being in each hut once, while both WSEB treatment replicates relevant to huts with PM IRS were in each hut twice.

**Table 1:** Experimental treatment arrangement allocation and rotation schedule

Rotation replicate day	Hut	Treatment
1	A	U1 + IRS
1	B	U3
1	C	U2 + IRS
1	D	T1
2	A	U2 + IRS
2	B	T2
2	C	U1 + IRS
2	D	U4
3	A	U1 + IRS
3	B	U4
3	C	U2 + IRS
3	D	T2
4	A	U2 + IRS
4	B	T1
4	C	U1 + IRS
4	D	U3

For indoor residual spraying (IRS) of pirimiphos-methyl (PM), as well as window screens and eave baffles (WSEBs) that were either untreated (U) or treated (T) with PM plus binding agent (BA) in the 4 experimental huts over each rotation replicate cycle of 4 days, with all huts being occupied each night by 2 adult male volunteers sleeping under a pyrethroid-treated long-lasting insecticidal net

U1, U2, U3, and U4 are WSEB-negative controls treated with BA only, while T1 and T2 are WSEBs treated with 2 g/sq m PM and BA

A round of 4 such rotation replicates of 4 nights were completed over a total of 16 nights. T1 and T2 were then hand washed ten times by immersing for 10 min in 8-l aliquots of distilled water containing 2 g/l of the same clothes washing soap used for the wire ball assays of wash resistance. This detergent was fully suspended just before washing the WSEB set and then rinsed twice for another 10 min in 8-l aliquots of distilled water, before being hung up to dry indoors for 12 h. These washed baffles were then re-evaluated in experimental huts over another 16 nights, comprising a full round of 4 rotation replicates of 4 nights. The WSEBs were then washed for a second sequence of 10 washes, as described above, and underwent a third 16-night round of experimental hut assessment.

The experimental evaluations of these insecticide-treated WSEBs were carried out between January and March 2017. IRS was applied to huts B and D on 10 January and the first round of 4 rotation replicates with unwashed PM-WSEBs was conducted between 14 and 29 January. The second round of 4 rotation replicates after the PM-WSEBs had been washed ten times were conducted between 6 and 22 February, while the third round after 20 washes was conducted between 28 and 15 March.

Every morning at 07.00, mosquitoes were collected with mouth aspirators from the exit traps placed behind half of the eaves and behind all of the windows, and from inside the hut using back pack aspirators. Mosquitoes which were already dead when collected were sorted and counted immediately, while those which were still alive were then kept in cups with access to sugar for 24 h in a humidified, ventilated, shaded field insectary. At the end of this holding period, live and dead mosquitoes were separated, sorted and counted. The cups holding mosquitoes from each of the three distinct collections (eave traps, window traps, and remaining indoors) were labelled by collection type, experimental hut identifier and mortality classification (dead upon collection, dead after 24 h, alive after 24 h). All anopheline mosquitoes were separated from culicines and then morphologically identified using taxonomic keys [27], sorted by sex and abdominal status, and then counted. All *Anopheles* mosquitoes caught were then desiccated over anhydrous calcium sulphate in microcentrifuge tubes, and stored at room temperature.

### Data collection, management and analysis

All field data were collected on hard copies of updated versions of the adult field collection experimental design (ED1) and sample sorting (SS3) forms, recently described for informatically robust collection of entomologic data [28]. To ensure compliance with the experimental design, all attributes defined by it were prefilled into the forms (Additional file 1). The effects of treatment (categorical independent variable) upon mosquito mortality were estimated separately for each 16-night round of replication, between which WSEBs were washed ten times each. The outcomes were estimated with GLMM using R software version 3.2.1. The experimental hut and day were treated as random effects, while the dependent variable (mortality, expressed as the cumulative proportion of mosquitoes which died in the huts or within 24 h of collection) was fitted with a logit link function and binomial distribution.

## Results

### Wire ball assessments with insectary-reared mosquitoes

**Fig. 2:**
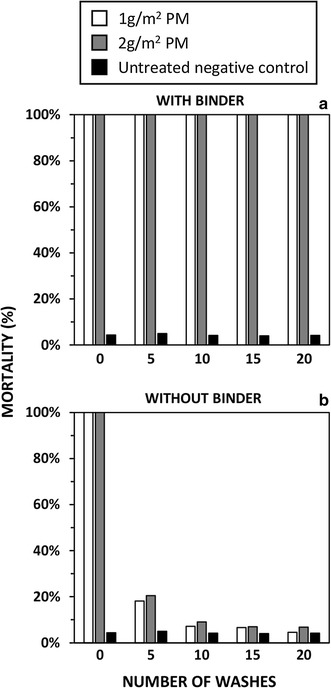
The efficacy of polyester netting treated with pirimiphos-methyl (PM) against pyrethroid-susceptible insectary-reared *Anopheles gambiae* in terms of mosquito mortality as measured with standardized wire ball assays with and without the binder

For the wire ball assays, 100% mortality was recorded for pyrethroid susceptible Kisumu strain 24 h after being exposed to either 1 or 2 g/sq m treatments with the binder, regardless of how many times they were washed. For treatments without the binder, 100% mortality was recorded at 0 washes and declined to less than 10% after 10 standard washes. Less than 5% mortality was consistently observed for the negative controls (Fig. 2).

### Experimental hut assessments against field populations of mosquitoes

A total of 2884 specimens from the *An. gambiae* complex were collected. The numbers of indoor-biting *An. funestus* were far lower than in most previous studies [22, 23, 29] so only 333 specimens from this group were obtained, probably because almost all households in the study area had recently been sprayed with PM through routine programmatic service delivery of IRS. Indeed *An. funestus* became very sparse towards the end of the study, resulting in < 10 specimens being caught per treatment group during the final round of experimental replication after 20 washes. Data for *An. funestus* were therefore excluded from the analysis and are not reported here.

Out of 300 specimens from the *An. gambiae* complex that were selected by randomly picking 100 mosquitoes from each experimental round for molecular identification to sibling species level, DNA from 265 (88%) were successfully amplified, out of which 258 (97.4%) were identified as *Anopheles arabiensis* and only 7 *(*2.6%) as *Anopheles quadriannulatus*. Therefore, no species-stratified analysis was considered necessary and results for total numbers from this complex are considered to be representative of *An. arabiensis*.

### Effects of IRS and WSEBs upon *Anopheles arabiensis*

**Fig. 3:**
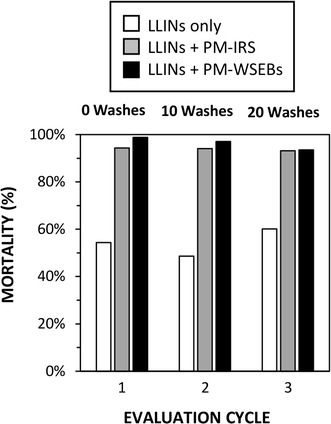
Shows the efficacy of window screens and eave baffles (WSEBs) treated with binding agent and 2 g/sq m pirimiphos-methyl (PM) or indoor residual spraying (IRS) with 1 g/sq m PM as supplementary vector control measures to pyrethroid-treated long-lasting insecticidal nets (LLINs), expressed in terms of mortality of *Anopheles arabiensis* entering experimental huts

In the first replication cycle after IRS application to 2 of the 4 huts, at which point the WSEBs had not yet been washed, both supplementary interventions using PM greatly increased mortality rates compared to pyrethroid-treated LLINs alone (Table 2, Fig. 3). When LLINs plus PM-treated WSEBs were compared with LLINs plus IRS with PM, mortality rates for the former appeared slightly superior (Odds ratio (OR) [95% confidence interval] = 4.3 [1.0, 18.7], P = 0.053). After 10 and 20 washes, mortality rates achieved by PM-WSEBs were indistinguishable from those for LLINs supplemented with relatively fresh PM-IRS that had been applied within the previous 2 months (P = 0.22 and 0.83, respectively).

**Table 2:** Mortality rates of *Anopheles arabiensis* in experimental huts

	Replication cycle 1 (WSEBs not washed)			Replication cycle 2 (WSEBs washed 10 times)			Replication cycle 3 (WSEBs washed 20 times)		
Treatments	Mortality [CI]^a^	OR^b^ [CI]^a^	P	Mortality [CI]^a^	OR^b^ [CI]^a^	P	Mortality [CI]^a^	OR^b^ [CI]^a^	P
*Anopheles arabiensis*									
LLINs only	0.58 [0.40,0.74]	1.00 NA^c^	NA^c^	0.57 [0.32,0.80]	1.00 NA^c^	NA^c^	0.50 [0.27,0.80]	1.00 NA^c^	NA^c^
LLINs + PM-IRS	0.96 [0.91,0.98]	16.3 [6.2,42.8]	< 0.001	0.95 [0.87,0.98]	15.3 [3.6,64.3]	< 0.001	0.95 [0.86,0.98]	18.8 [4.3,77.5]	< 0.001
LLINs + PM-WSEBs	0.99 [0.96,1.00]	70.0 [21.8224]	< 0.001	0.98 [0.94,1.00]	41.7 [19.8,87.6]	< 0.001	0.94 [0.79,0.98]	15.3 [4.5,51.5]	< 0.001

Occupied by volunteers sleeping under long-lasting insecticidal nets (LLINs) treated with deltamethrin (a pyrethroid) as used alone, supplemented with indoor residual spraying (IRS) of pirimiphos-methyl (PM, an organophosphate), or supplemented with window screens and eave baffles (WSEBs) treated with PM plus a binding agent

^a^95% Confidence interval

^b^Odds ratio

^c^Not applicable

## Discussion

Overall, LLINs supplemented with WSEBs, co-treated with PM and BA, killed mosquitoes in at least equal proportions to LLINs supplemented with conventional IRS using the same organophosphate active ingredient, and at least matched the WHO wash-resistance requirements for pyrethroid-treatment of LLINs. While this study is limited in that it did not use a complete Latin Squares design, because IRS was sprayed in two huts and cannot be rotated like LLINs and WSEBS, mortality is a robust binary outcome less likely to vary with hut because these were of a standardized design [25, 26] and any variations in vector density will be reflected in both the nominator and the denominator. Taken at face value, these results indicate that PM plus BA co-treatments of WSEBs could potentially last for up to 3 years, assuming that 20 standard washes stipulated by WHO pesticide evaluation scheme guidelines for LLINs are indeed representative of 3 years of field use [19]. If such durability could be realized under conditions of routine use, insecticide retreatment frequency could be reduced relative to IRS applications, which typically last between 2 and 9 months on highly variable wall and ceiling surfaces [30–32]. Combined with the reduced surface area that needs to be treated when PM is selectively applied to WSEBs, instead of entire inner surfaces of houses, this suggests that insecticide consumption requirements could indeed be reduced by at least an order of magnitude [17]. Furthermore, such reduced retreatment frequency relative to IRS, which requires full access to the insides of houses and temporary removal of furniture and food once or twice a year, could also mitigate against householder compliance fatigue and refusals [33–35]. The potential for in situ application by brush or roller, rather than pre-installation dipping, may also have significant advantages over spraying in terms of safety, convenience and acceptability.

Nevertheless, these results should be interpreted with caution, because the WSEBs used here were just an experimental prototype that can only be used under the artificial conditions of standardized experimental huts. Developing scalable procedures for installation in a range of different housing designs may be challenging, and experience with traditional house screening suggests that cost represents a significant obstacle [36]. The practicality, effectiveness and costs of such scalable formats will then need to be rigorously evaluated under representative field conditions before programmatic scale up could be considered. Also, long-term durability in the field under representative conditions of routine use in regular houses will have to be assessed, probably with netting materials that are far more robust than polyester. While demonstration of wash resistance is encouraging, this is not the same thing as normal exposure to wind, rain, sunlight, and routine wear-and-tear in real houses.

Like PM-IRS, supplementation of pyrethroid-treated LLINs with PM-WSEBs had far greater impacts upon pyrethroid-resistant field population of *An. arabiensis*, than LLINs alone. In areas where insecticide resistance has emerged WSEBs treated with complementary insecticides may have potential for cost-effectively restoring full impact of indoor-based malaria vector control [8, 37, 38]. Additionally WSEBs may counteract some of the limitations of LLINs against behaviourally resilient and/or resistant vectors, such as endophagic but early-exiting *An. arabiensis* [39, 40]. Indeed it is noteworthy that *An. arabiensis* constituted a far greater proportion of the *An. gambiae* complex in this setting at the time of this study than before the roll out of PM-IRS [13, 22]. The vector system against which these WSEBs were so efficacious was therefore probably a representative example of residual transmission by behaviourally resistant/resilient vectors.

## Conclusion

WSEBs may remain insecticidal for years rather than months. Insecticide consumption needs for WSEBs could therefore be far lower than for IRS, because it may be possible to not only reduce surface area to be treated in each house, but also the re-treatment frequency. This study therefore adds further evidence that WSEBs could have several complementary benefits, in terms of incremental impacts upon malaria transmission and improved, more affordable resistance management, if they could be implemented at scale alongside LLINs. However, for programmatic vector control, new WSEBs prototypes will need to be developed that can be practically and affordably installed in a diversity of housing designs. If such programmatically effective WSEBs can be designed, they could be used to counteract the low and declining coverage of IRS with expensive alternatives to pyrethroids [10, 11] across Africa and beyond.

## Additional file

**Additional file 1.** Data collection forms for the experimental hut study, South East of Zambia.

## Abbreviations

BA: binding agent
GLMM: generalized linear mixed models
GPIRM: global plan for insecticide resistance management
IRS: indoor residual spraying
LLINs: long-lasting insecticidal nets
PM: pirimiphos-methyl
T in Table 1: treatment
U in Table 2: untreated
WHO: World Health Organization
WSEBs: window screens and eave baffles
