# Supplementary material for: Wash-resistance of pirimiphos-methyl insecticide treatments of window screens and eave baffles for killing indoor-feeding malaria vector mosquitoes: an experimental hut trial, South East of Zambia
Source: Malar J. 2018 Apr 13;17:164. doi: 10.1186/s12936-018-2309-2 (PMC5899344; doi:10.1186/s12936-018-2309-2)
Supplement: Supplementary file 1 — Additional file 1. Data collection forms for the experimental hut study, South East of Zambia. [file 12936_2018_2309_MOESM1_ESM.pdf]

Experimental design: Field Collections	PROJECT CODE (PC): WT	EXPERIMENT NO (EN): 1	SERIAL NO (SEN): 1068
	FORM TYPE (FT): ED1	SITE (SI): Chisobe	

COLLECTION ATTRUBUTES — GENERIC: columns C–I; EXPERIMENT SPECIFIC: columns J–U

MARK X FOR ALL VARIABLES WHICH MUST BE RECORDED

Form Row (FR)	Date of Collection (DT)	Enumeration area (EA)	Cluster (CR)	Compound or Plot (CP)	Household (HH)	Structure/Habitat ID (SID)	Method (ME)	Indoor/Outdoor (IN) (1 = in, 2 = out)	Habitat type (HT)	Start Time (ST)	Finish Time (FT)	Holding Period (HP)	Round (RND)	Block (BLK)	House/Hut (SHH)	Station (ST)	Volunteer initials (VI)	Treatment (TR)	Experimental Day (DY)	No. Household Sampled (HS)	Valid Catch (VC): 1=yes, 2=missing data	SS Form Serial No. (DSEN)	Notes
							X	X		X	X												
A	B	C	D	E	F	G	H	I	J	K	L	M	N	O	P	Q	R	S	T	U	V	W	X
01	15/17/2017	Chisobe					5	1		19	7				A		GC/TM	U1 + IRS	1		1	0005	
02	15/17/2017	Chisobe					10	1		19	7				A		GC/TM	U1 + IRS	1		1	0006	
03	15/17/2017	Chisobe					11	1		19	7				A		GC/TM	U1 + IRS	1		1	0007	
04	15/17/2017	Chisobe					5	1		19	7				B		RZ/BT	U3	1		1	0008	
05	15/17/2017	Chisobe					10	1		19	7				B		RZ/BT	U3	1		1	0009	
06	15/17/2017	Chisobe					11	1		19	7				B		RZ/BT	U3	1		1	0010	
07	15/17/2017	Chisobe					5	1		19	7				C		MZ/OZ	U2 + IRS	1		1	0012	
08	15/17/2017	Chisobe					10	1		19	7				C		MZ/OZ	U2 + IRS	1		1	0013	
09	15/17/2017	Chisobe					11	1		19	7				C		MZ/OZ	U2 + IRS	1		1	0014	
10	15/17/2017	Chisobe					5	1		19	7				D		BN/KN	T1	1		1	0015	
11	15/17/2017	Chisobe					10	1		19	7				D		BN/KN	T1	1		1	0016	
12	15/17/2017	Chisobe					11	1		19	7				D		BN/KN	T1	1		1	0017	
13																							
14																							
15																							
16																							
17																							
18																							
19																							
20																							

EXPERIMENT SUPERVISORS INITIALS (ESI): D.C	AND SIGNATURE:
RESPONSIBLE SCIENTISTS INITIALS (RSI:) D.C	AND SIGNATURE:

Method (ME): 01 = HLC; 02 = ITT; 03 = CDC LT; 04 = Resting box; 05 = Aspirator resting sites; 06 = Floor; 07 = Entry window; 08 = Entry eave; 09 = Entry; 10 = Exit window; 11 = Exit eave; 12 = Exit; 13 = PSC; 14 = Dip; 15 = Substrate; 16 = Emergence trap; 17 = Ovitrap
Habitat type (HT): 01 = Puddles & tire tracks; 02 = Swampy areas; 03 = Mangrove swamp/saltwater marsh; 04 = Drain/ditch; 05 = Construction pit/foundations/man-made hole; 06 = Water storage container; 07 = Rice paddy; 08 = Raised bed agriculture; 09 = Other agriculture; 10 = Stream/river bed; 11 = Pond; 12 = Other (describe)

Sample Sorting: Batch and/or Pool	Project Code (PC): Avecnet WP2	Experiment No. (EN): 1	SERIAL NO. (SEN): 0005
FORM TYPE (FT): SS3	ED Form Serial No. (SSEN): 1068	ED Form Row (SFR): 1	

Form Row (FR)	Dead (DD) (1 = yes, 2 = no)	Taxon (TX)	Sex and Abdominal Status (SAS)	Mated (MT) (1 = yes, 2 = no)	Choice (CH)	Label (LA)	Finish Date (FD)	Finish Time (FT)	Body Form (BF)	Holding period (HP)	Other 5 (OTH5)	Other 6 (OTH6)	Other 7 (OTH7)	No. Observed (N)	Sample Lable Code (SLC)	No. of Individuals	Number of batches	Number in Batch 1 (SID = 01)	Number in Batch 2 (SID = 02)	Number in Batch 3 (SID = 03)	Number in Batch 4 (SID = 04)	Number in Batch 5 (SID = 05)	Number in Batch 6 (SID = 06)	Number in Batch 7 (SID = 07)	Number Discarded
	Sorting/Obersvation Variables															Sample type variables									
	MARK X FOR ALL VARIABLES WHICH MUST BE RECORDED																								
	x	x	x																						
	Define whether the attribute is categorical (CA) or continuous (CO)															Sample type (ST):									
A	B	C	D	E	F	G	H	I	J	K	L	M	N	O	P	Q	R	S	T	U	V	W	X	Y	Z
																1		2	2	2	2	2	2	2	
01	1	1	2					7	1	0															
02	1	1	3					7	1	0				7	S013	1									
03	1	1	4					7	1	0															
04	1	1	5					7	1	0															
05	1	2	2					7	1	0				[illegible]	[illegible]	[illegible]									
06	1	2	3					7	1	0															
07	1	2	4					7	1	0															
08	1	2	5					7	1	0															
09	1	1	2					7	1	24															
10	1	1	3					7	1	24															
11	1	1	4					7	1	24															
12	1	1	5					7	1	24															
13	1	2	2					7	1	24															
14	1	2	3					7	1	24															
15	1	2	4					7	1	24															
16	1	2	5					7	1	24															
17	2	1	2					7	1	24															
18	2	1	3					7	1	24															
19	2	1	4					7	1	24															
20	2	1	5					7	1	24															
21	2	2	2					7	1	24															
22	2	2	3					7	1	24															
23	2	2	4					7	1	24															
24	2	2	5					7	1	24															

Sex and Abdominal Status (SAS): 1 = Total Male; 2 = Unfed; 3 = Partly Fed; 4 = Fed; 5 = Gravid/semigravid; 6 = Total Female
Body Part (BP): 01 = Whole Adult; 13 = Eggs; 16 = Larvae; 17 = L1; 18 = L2; 19 = L3; 20 = L4; 21 = L1&L2; 22 = L3&L4; 23 = Pupae

Labelling details: First line: FT.SEN.FR
Second line: SLC.BP.ST.SID

Sample Sorting: Batch and/or Pool	Project Code (PC): Avecnet WP2	Experiment No. (EN): 1	SERIAL NO. (SEN): 0006
FORM TYPE (FT): SS3	ED Form Serial No. (SSEN): 1068	ED Form Row (SFR): 2	

Sorting/Obersvation Variables | Sample type variables

Form Row (FR)	Dead (DD) (1 = yes, 2 = no)	Taxon (TX)	Sex and Abdominal Status (SAS)	Mated (MT) (1 = yes, 2 = no)	Choice (CH)	Label (LA)	Finish Date (FD)	Finish Time (FT)	Body Form (BF)	Holding period (HP)	Other 5 (OTH5)	Other 6 (OTH6)	Other 7 (OTH7)	No. Observed (N)	Sample Lable Code (SLC)	No. of Individuals	Number of batches	Number in Batch 1 (SID = 01)	Number in Batch 2 (SID = 02)	Number in Batch 3 (SID = 03)	Number in Batch 4 (SID = 04)	Number in Batch 5 (SID = 05)	Number in Batch 6 (SID = 06)	Number in Batch 7 (SID = 07)	Number Discarded
MARK X FOR ALL VARIABLES WHICH MUST BE RECORDED	x	x	x																						
Define whether the attribute is categorical (CA) or continuous (CO)																Sample type (ST):									
A	B	C	D	E	F	G	H	I	J	K	L	M	N	O	P	Q	R	S	T	U	V	W	X	Y	Z
																1		2	2	2	2	2	2	2	
01	1	1	2					7	1	0															
02	1	1	3					7	1	0				1	5014	1									
03	1	1	4					7	1	0															
04	1	1	5					7	1	0															
05	1	2	2					7	1	0															
06	1	2	3					7	1	0															
07	1	2	4					7	1	0															
08	1	2	5					7	1	0															
09	1	1	2					7	1	24															
10	1	1	3					7	1	24															
11	1	1	4					7	1	24															
12	1	1	5					7	1	24															
13	1	2	2					7	1	24															
14	1	2	3					7	1	24															
15	1	2	4					7	1	24															
16	1	2	5					7	1	24															
17	2	1	2					7	1	24															
18	2	1	3					7	1	24															
19	2	1	4					7	1	24															
20	2	1	5					7	1	24															
21	2	2	2					7	1	24															
22	2	2	3					7	1	24															
23	2	2	4					7	1	24															
24	2	2	5					7	1	24															

Sex and Abdominal Status (SAS): 1 = Total Male; 2 = Unfed; 3 = Partly Fed; 4 = Fed; 5 = Gravid/semigravid; 6 = Total Female
Body Part (BP): 01 = Whole Adult; 13 = Eggs; 16 = Larvae; 17 = L1; 18 = L2; 19 = L3; 20 = L4; 21 = L1&L2; 22 = L3&L4; 23 = Pupae

Labelling details: First line: FT.SEN.FR
Second line: SLC.BP.ST.SID

Sample Sorting: Batch and/or Pool	Project Code (PC): Avecnet WP2	Experiment No. (EN): 1	SERIAL NO. (SEN): 0007
FORM TYPE (FT): SS3	ED Form Serial No. (SSEN): 1068	ED Form Row (SFR): 3	

Sorting/Obersvation Variables; Sample type variables

Form Row (FR)	Dead (DD) (1 = yes, 2 = no)	Taxon (TX)	Sex and Abdominal Status (SAS)	Mated (MT) (1 = yes, 2 = no)	Choice (CH)	Label (LA)	Finish Date (FD)	Finish Time (FT)	Body Form (BF)	Holding period (HP)	Other 5 (OTH5)	Other 6 (OTH6)	Other 7 (OTH7)	No. Observed (N)	Sample Lable Code (SLC)	No. of Individuals	Number of batches	Number in Batch 1 (SID = 01)	Number in Batch 2 (SID = 02)	Number in Batch 3 (SID = 03)	Number in Batch 4 (SID = 04)	Number in Batch 5 (SID = 05)	Number in Batch 6 (SID = 06)	Number in Batch 7 (SID = 07)	Number Discarded
MARK X FOR ALL VARIABLES WHICH MUST BE RECORDED																									
	x	x	x																						
Define whether the attribute is categorical (CA) or continuous (CO)																Sample type (ST):									
A	B	C	D	E	F	G	H	I	J	K	L	M	N	O	P	Q	R	S	T	U	V	W	X	Y	Z
																1		2	2	2	2	2	2	2	
01	1	1	2					7	1	0															
02	1	1	3					7	1	0				9	5014	1									
03	1	1	4					7	1	0															
04	1	1	5					7	1	0															
05	1	2	2					7	1	0															
06	1	2	3					7	1	0															
07	1	2	4					7	1	0															
08	1	2	5					7	1	0															
09	1	1	2					7	1	24															
10	1	1	3					7	1	24															
11	1	1	4					7	1	24															
12	1	1	5					7	1	24															
13	1	2	2					7	1	24															
14	1	2	3					7	1	24															
15	1	2	4					7	1	24															
16	1	2	5					7	1	24															
17	2	1	2					7	1	24															
18	2	1	3					7	1	24															
19	2	1	4					7	1	24															
20	2	1	5					7	1	24															
21	2	2	2					7	1	24															
22	2	2	3					7	1	24															
23	2	2	4					7	1	24															
24	2	2	5					7	1	24															

Sex and Abdominal Status (SAS): 1 = Total Male; 2 = Unfed; 3 = Partly Fed; 4 = Fed; 5 = Gravid/semigravid; 6 = Total Female
Body Part (BP): 01 = Whole Adult; 13 = Eggs; 16 = Larvae; 17 = L1; 18 = L2; 19 = L3; 20 = L4; 21 = L1&L2; 22 = L3&L4; 23 = Pupae

Labelling details: First line: FT.SEN.FR
Second line: SLC.BP.ST.SID

Sample Sorting: Batch and/or Pool	Project Code (PC): Avecnet WP2	Experiment No. (EN): 1	SERIAL NO. (SEN): 0008
FORM TYPE (FT): SS3	ED Form Serial No. (SSEN): 1068	ED Form Row (SFR): 4	

Sorting/Obersvation Variables — Sample type variables

Form Row (FR)	Dead (DD) (1 = yes, 2 = no)	Taxon (TX)	Sex and Abdominal Status (SAS)	Mated (MT) (1 = yes, 2 = no)	Choice (CH)	Label (LA)	Finish Date (FD)	Finish Time (FT)	Body Form (BF)	Holding period (HP)	Other 5 (OTH5)	Other 6 (OTH6)	Other 7 (OTH7)	No. Observed (N)	Sample Lable Code (SLC)	No. of Individuals	Number of batches	Number in Batch 1 (SID = 01)	Number in Batch 2 (SID = 02)	Number in Batch 3 (SID = 03)	Number in Batch 4 (SID = 04)	Number in Batch 5 (SID = 05)	Number in Batch 6 (SID = 06)	Number in Batch 7 (SID = 07)	Number Discarded
MARK X FOR ALL VARIABLES WHICH MUST BE RECORDED	x	x	x																						
Define whether the attribute is categorical (CA) or continuous (CO)																									
Sample type (ST):																1		2	2	2	2	2	2	2	
A	B	C	D	E	F	G	H	I	J	K	L	M	N	O	P	Q	R	S	T	U	V	W	X	Y	Z
01	1	1	2					7	1	0															
02	1	1	3					7	1	0				3	5015	1									
03	1	1	4					7	1	0															
04	1	1	5					7	1	0															
05	1	2	2					7	1	0															
06	1	2	3					7	1	0															
07	1	2	4					7	1	0															
08	1	2	5					7	1	0															
09	1	1	2					7	1	24															
10	1	1	3					7	1	24															
11	1	1	4					7	1	24															
12	1	1	5					7	1	24															
13	1	2	2					7	1	24															
14	1	2	3					7	1	24															
15	1	2	4					7	1	24															
16	1	2	5					7	1	24															
17	2	1	2					7	1	24															
18	2	1	3					7	1	24															
19	2	1	4					7	1	24															
20	2	1	5					7	1	24															
21	2	2	2					7	1	24															
22	2	2	3					7	1	24															
23	2	2	4					7	1	24															
24	2	2	5					7	1	24															

Sex and Abdominal Status (SAS): 1 = Total Male; 2 = Unfed; 3 = Partly Fed; 4 = Fed; 5 = Gravid/semigravid; 6 = Total Female
Body Part (BP): 01 = Whole Adult; 13 = Eggs; 16 = Larvae; 17 = L1; 18 = L2; 19 = L3; 20 = L4; 21 = L1&L2; 22 = L3&L4; 23 = Pupae

Labelling details: First line: FT.SEN.FR
Second line: SLC.BP.ST.SID

Sample Sorting: Batch and/or Pool	Project Code (PC): Avecnet WP2	Experiment No. (EN): 1	SERIAL NO. (SEN): 0009
FORM TYPE (FT): SS3	ED Form Serial No. (SSEN): 1068	ED Form Row (SFR): 5	

Form Row (FR)	Dead (DD) (1 = yes, 2 = no)	Taxon (TX)	Sex and Abdominal Status (SAS)	Mated (MT) (1 = yes, 2 = no)	Choice (CH)	Label (LA)	Finish Date (FD)	Finish Time (FT)	Body Form (BF)	Holding period (HP)	Other 5 (OTH5)	Other 6 (OTH6)	Other 7 (OTH7)	No. Observed (N)	Sample Lable Code (SLC)	No. of Individuals	Number of batches	Number in Batch 1 (SID = 01)	Number in Batch 2 (SID = 02)	Number in Batch 3 (SID = 03)	Number in Batch 4 (SID = 04)	Number in Batch 5 (SID = 05)	Number in Batch 6 (SID = 06)	Number in Batch 7 (SID = 07)	Number Discarded
	Sorting/Obersvation Variables															Sample type variables									
	MARK X FOR ALL VARIABLES WHICH MUST BE RECORDED																								
	x	x	x																						
	Define whether the attribute is categorical (CA) or continuous (CO)															Sample type (ST):									
																1		2	2	2	2	2	2	2	
A	B	C	D	E	F	G	H	I	J	K	L	M	N	O	P	Q	R	S	T	U	V	W	X	Y	Z
01	1	1	2					7	1	0															
02	1	1	3					7	1	0				6	5016	1									
03	1	1	4					7	1	0															
04	1	1	5					7	1	0															
05	1	2	2					7	1	0															
06	1	2	3					7	1	0															
07	1	2	4					7	1	0															
08	1	2	5					7	1	0															
09	1	1	2					7	1	24															
10	1	1	3					7	1	24															
11	1	1	4					7	1	24															
12	1	1	5					7	1	24															
13	1	2	2					7	1	24															
14	1	2	3					7	1	24															
15	1	2	4					7	1	24															
16	1	2	5					7	1	24															
17	2	1	2					7	1	24				[illegible]											
18	2	1	3					7	1	24				1	5017	1									
19	2	1	4					7	1	24															
20	2	1	5					7	1	24															
21	2	2	2					7	1	24															
22	2	2	3					7	1	24															
23	2	2	4					7	1	24				2	5018	1									
24	2	2	5					7	1	24															

Sex and Abdominal Status (SAS): 1 = Total Male; 2 = Unfed; 3 = Partly Fed; 4 = Fed; 5 = Gravid/semigravid; 6 = Total Female
Body Part (BP): 01 = Whole Adult; 13 = Eggs; 16 = Larvae; 17 = L1; 18 = L2; 19 = L3; 20 = L4; 21 = L1&L2; 22 = L3&L4; 23 = Pupae

Labelling details: First line: FT.SEN.FR
Second line: SLC.BP.ST.SID

Sample Sorting: Batch and/or Pool	Project Code (PC): Avecnet WP2	Experiment No. (EN): 1	SERIAL NO. (SEN): 0010
FORM TYPE (FT): SS3	ED Form Serial No. (SSEN): 1068	ED Form Row (SFR): 6	

Form Row (FR)	Dead (DD) (1 = yes, 2 = no)	Taxon (TX)	Sex and Abdominal Status (SAS)	Mated (MT) (1 = yes, 2 = no)	Choice (CH)	Label (LA)	Finish Date (FD)	Finish Time (FT)	Body Form (BF)	Holding period (HP)	Other 5 (OTH5)	Other 6 (OTH6)	Other 7 (OTH7)	No. Observed (N)	Sample Lable Code (SLC)	No. of Individuals	Number of batches	Number in Batch 1 (SID = 01)	Number in Batch 2 (SID = 02)	Number in Batch 3 (SID = 03)	Number in Batch 4 (SID = 04)	Number in Batch 5 (SID = 05)	Number in Batch 6 (SID = 06)	Number in Batch 7 (SID = 07)	Number Discarded
	Sorting/Obersvation Variables															Sample type variables									
	MARK X FOR ALL VARIABLES WHICH MUST BE RECORDED																								
	x	x	x																						
	Define whether the attribute is categorical (CA) or continuous (CO)															Sample type (ST):									
A	B	C	D	E	F	G	H	I	J	K	L	M	N	O	P	Q	R	S	T	U	V	W	X	Y	Z
																1		2	2	2	2	2	2	2	
01	1	1	2					7	1	0															
02	1	1	3					7	1	0				4	5019	1									
03	1	1	4					7	1	0															
04	1	1	5					7	1	0															
05	1	2	2					7	1	0															
06	1	2	3					7	1	0															
07	1	2	4					7	1	0															
08	1	2	5					7	1	0															
09	1	1	2					7	1	24															
10	1	1	3					7	1	24															
11	1	1	4					7	1	24															
12	1	1	5					7	1	24															
13	1	2	2					7	1	24															
14	1	2	3					7	1	24															
15	1	2	4					7	1	24															
16	1	2	5					7	1	24															
17	2	1	2					7	1	24															
18	2	1	3					7	1	24															
19	2	1	4					7	1	24															
20	2	1	5					7	1	24															
21	2	2	2					7	1	24															
22	2	2	3					7	1	24															
23	2	2	4					7	1	24															
24	2	2	5					7	1	24															

Sex and Abdominal Status (SAS): 1 = Total Male; 2 = Unfed; 3 = Partly Fed; 4 = Fed; 5 = Gravid/semigravid; 6 = Total Female
Body Part (BP): 01 = Whole Adult; 13 = Eggs; 16 = Larvae; 17 = L1; 18 = L2; 19 = L3; 20 = L4; 21 = L1&L2; 22 = L3&L4; 23 = Pupae

Labelling details: First line: FT.SEN.FR
Second line: SLC.BP.ST.SID

Sample Sorting: Batch and/or Pool	Project Code (PC): Avecnet WP2	Experiment No. (EN): 1	SERIAL NO. (SEN): 0012
FORM TYPE (FT): SS3	ED Form Serial No. (SSEN): 1068	ED Form Row (SFR): 7	

Form Row (FR)	Dead (DD) (1 = yes, 2 = no)	Taxon (TX)	Sex and Abdominal Status (SAS)	Mated (MT) (1 = yes, 2 = no)	Choice (CH)	Label (LA)	Finish Date (FD)	Finish Time (FT)	Body Form (BF)	Holding period (HP)	Other 5 (OTH5)	Other 6 (OTH6)	Other 7 (OTH7)	No. Observed (N)	Sample Lable Code (SLC)	No. of Individuals	Number of batches	Number in Batch 1 (SID = 01)	Number in Batch 2 (SID = 02)	Number in Batch 3 (SID = 03)	Number in Batch 4 (SID = 04)	Number in Batch 5 (SID = 05)	Number in Batch 6 (SID = 06)	Number in Batch 7 (SID = 07)	Number Discarded
MARK X FOR ALL VARIABLES WHICH MUST BE RECORDED	x	x	x																						
Define whether the attribute is categorical (CA) or continuous (CO)																									
Sample type (ST):																1		2	2	2	2	2	2	2	
A	B	C	D	E	F	G	H	I	J	K	L	M	N	O	P	Q	R	S	T	U	V	W	X	Y	Z
01	1	1	2					7	1	0															
02	1	1	3					7	1	0				4	5020	1									
03	1	1	4					7	1	0															
04	1	1	5					7	1	0															
05	1	2	2					7	1	0															
06	1	2	3					7	1	0															
07	1	2	4					7	1	0															
08	1	2	5					7	1	0															
09	1	1	2					7	1	24															
10	1	1	3					7	1	24															
11	1	1	4					7	1	24															
12	1	1	5					7	1	24															
13	1	2	2					7	1	24															
14	1	2	3					7	1	24															
15	1	2	4					7	1	24															
16	1	2	5					7	1	24															
17	2	1	2					7	1	24															
18	2	1	3					7	1	24				3	5021	1									
19	2	1	4					7	1	24															
20	2	1	5					7	1	24															
21	2	2	2					7	1	24															
22	2	2	3					7	1	24															
23	2	2	4					7	1	24															
24	2	2	5					7	1	24															

Sorting/Obersvation Variables — columns B–N; Sample type variables — columns Q–Z.

Sex and Abdominal Status (SAS): 1 = Total Male; 2 = Unfed; 3 = Partly Fed; 4 = Fed; 5 = Gravid/semigravid; 6 = Total Female
Body Part (BP): 01 = Whole Adult; 13 = Eggs; 16 = Larvae; 17 = L1; 18 = L2; 19 = L3; 20 = L4; 21 = L1&L2; 22 = L3&L4; 23 = Pupae

Labelling details: First line: FT.SEN.FR
Second line: SLC.BP.ST.SID

Sample Sorting: Batch and/or Pool	Project Code (PC): Avecnet WP2	Experiment No. (EN): 1	SERIAL NO. (SEN): 0013
FORM TYPE (FT): SS3	ED Form Serial No. (SSEN): 1068	ED Form Row (SFR): 8	

Form Row (FR)	Dead (DD) (1 = yes, 2 = no)	Taxon (TX)	Sex and Abdominal Status (SAS)	Mated (MT) (1 = yes, 2 = no)	Choice (CH)	Label (LA)	Finish Date (FD)	Finish Time (FT)	Body Form (BF)	Holding period (HP)	Other 5 (OTH5)	Other 6 (OTH6)	Other 7 (OTH7)	No. Observed (N)	Sample Lable Code (SLC)	No. of Individuals	Number of batches	Number in Batch 1 (SID = 01)	Number in Batch 2 (SID = 02)	Number in Batch 3 (SID = 03)	Number in Batch 4 (SID = 04)	Number in Batch 5 (SID = 05)	Number in Batch 6 (SID = 06)	Number in Batch 7 (SID = 07)	Number Discarded
MARK X FOR ALL VARIABLES WHICH MUST BE RECORDED	x	x	x													Sample type (ST):									
Define whether the attribute is categorical (CA) or continuous (CO)																									
A	B	C	D	E	F	G	H	I	J	K	L	M	N	O	P	Q	R	S	T	U	V	W	X	Y	Z
																1		2	2	2	2	2	2	2	
01	1	1	2					7	1	0															
02	1	1	3					7	1	0				1	5022	1									
03	1	1	4					7	1	0															
04	1	1	5					7	1	0															
05	1	2	2					7	1	0															
06	1	2	3					7	1	0															
07	1	2	4					7	1	0															
08	1	2	5					7	1	0															
09	1	1	2					7	1	24															
10	1	1	3					7	1	24															
11	1	1	4					7	1	24															
12	1	1	5					7	1	24															
13	1	2	2					7	1	24															
14	1	2	3					7	1	24															
15	1	2	4					7	1	24															
16	1	2	5					7	1	24															
17	2	1	2					7	1	24															
18	2	1	3					7	1	24															
19	2	1	4					7	1	24															
20	2	1	5					7	1	24															
21	2	2	2					7	1	24															
22	2	2	3					7	1	24															
23	2	2	4					7	1	24															
24	2	2	5					7	1	24															

Sex and Abdominal Status (SAS): 1 = Total Male; 2 = Unfed; 3 = Partly Fed; 4 = Fed; 5 = Gravid/semigravid; 6 = Total Female
Body Part (BP): 01 = Whole Adult; 13 = Eggs; 16 = Larvae; 17 = L1; 18 = L2; 19 = L3; 20 = L4; 21 = L1&L2; 22 = L3&L4; 23 = Pupae

Labelling details: First line: FT.SEN.FR
Second line: SLC.BP.ST.SID

Sample Sorting: Batch and/or Pool	Project Code (PC): Avecnet WP2	Experiment No. (EN): 1	SERIAL NO. (SEN): 0014
FORM TYPE (FT): SS3	ED Form Serial No. (SSEN): 1068	ED Form Row (SFR): 9	

Form Row (FR)	Dead (DD) (1 = yes, 2 = no)	Taxon (TX)	Sex and Abdominal Status (SAS)	Mated (MT) (1 = yes, 2 = no)	Choice (CH)	Label (LA)	Finish Date (FD)	Finish Time (FT)	Body Form (BF)	Holding period (HP)	Other 5 (OTH5)	Other 6 (OTH6)	Other 7 (OTH7)	No. Observed (N)	Sample Lable Code (SLC)	No. of Individuals	Number of batches	Number in Batch 1 (SID = 01)	Number in Batch 2 (SID = 02)	Number in Batch 3 (SID = 03)	Number in Batch 4 (SID = 04)	Number in Batch 5 (SID = 05)	Number in Batch 6 (SID = 06)	Number in Batch 7 (SID = 07)	Number Discarded
	Sorting/Obersvation Variables															Sample type variables									
	MARK X FOR ALL VARIABLES WHICH MUST BE RECORDED																								
	x	x	x																						
	Define whether the attribute is categorical (CA) or continuous (CO)															Sample type (ST):									
A	B	C	D	E	F	G	H	I	J	K	L	M	N	O	P	Q	R	S	T	U	V	W	X	Y	Z
																1		2	2	2	2	2	2	2	
01	1	1	2					7	1	0															
02	1	1	3					7	1	0				2	5023	1									
03	1	1	4					7	1	0															
04	1	1	5					7	1	0				[illegible]											
05	1	2	2					7	1	0															
06	1	2	3					7	1	0				1	5024	1									
07	1	2	4					7	1	0															
08	1	2	5					7	1	0															
09	1	1	2					7	1	24															
10	1	1	3					7	1	24															
11	1	1	4					7	1	24															
12	1	1	5					7	1	24															
13	1	2	2					7	1	24															
14	1	2	3					7	1	24															
15	1	2	4					7	1	24															
16	1	2	5					7	1	24															
17	2	1	2					7	1	24															
18	2	1	3					7	1	24															
19	2	1	4					7	1	24															
20	2	1	5					7	1	24															
21	2	2	2					7	1	24															
22	2	2	3					7	1	24															
23	2	2	4					7	1	24															
24	2	2	5					7	1	24															

Sex and Abdominal Status (SAS): 1 = Total Male; 2 = Unfed; 3 = Partly Fed; 4 = Fed; 5 = Gravid/semigravid; 6 = Total Female
Body Part (BP): 01 = Whole Adult; 13 = Eggs; 16 = Larvae; 17 = L1; 18 = L2; 19 = L3; 20 = L4; 21 = L1&L2; 22 = L3&L4; 23 = Pupae

Labelling details: First line: FT.SEN.FR
Second line: SLC.BP.ST.SID

Sample Sorting: Batch and/or Pool	Project Code (PC): Avecnet WP2	Experiment No. (EN): 1	SERIAL NO. (SEN): 0015
FORM TYPE (FT): SS3	ED Form Serial No. (SSEN): 1068	ED Form Row (SFR): 16	

Form Row (FR)	Sorting/Obersvation Variables															Sample type variables									
	Dead (DD) (1 = yes, 2 = no)	Taxon (TX)	Sex and Abdominal Status (SAS)	Mated (MT) (1 = yes, 2 = no)	Choice (CH)	Label (LA)	Finish Date (FD)	Finish Time (FT)	Body Form (BF)	Holding period (HP)	Other 5 (OTH5)	Other 6 (OTH6)	Other 7 (OTH7)	No. Observed (N)	Sample Lable Code (SLC)	No. of Individuals	Number of batches	Number in Batch 1 (SID = 01)	Number in Batch 2 (SID = 02)	Number in Batch 3 (SID = 03)	Number in Batch 4 (SID = 04)	Number in Batch 5 (SID = 05)	Number in Batch 6 (SID = 06)	Number in Batch 7 (SID = 07)	Number Discarded
	MARK X FOR ALL VARIABLES WHICH MUST BE RECORDED																								
	x	x	x																						
	Define whether the attribute is categorical (CA) or continuous (CO)															Sample type (ST):									
A	B	C	D	E	F	G	H	I	J	K	L	M	N	O	P	Q	R	S	T	U	V	W	X	Y	Z
																1		2	2	2	2	2	2	2	
01	1	1	2					7	1	0															
02	1	1	3					7	1	0				0											
03	1	1	4					7	1	0															
04	1	1	5					7	1	0															
05	1	2	2					7	1	0															
06	1	2	3					7	1	0				0											
07	1	2	4					7	1	0															
08	1	2	5					7	1	0															
09	1	1	2					7	1	24															
10	1	1	3					7	1	24															
11	1	1	4					7	1	24															
12	1	1	5					7	1	24															
13	1	2	2					7	1	24															
14	1	2	3					7	1	24															
15	1	2	4					7	1	24															
16	1	2	5					7	1	24															
17	2	1	2					7	1	24				0											
18	2	1	3					7	1	24															
19	2	1	4					7	1	24															
20	2	1	5					7	1	24															
21	2	2	2					7	1	24															
22	2	2	3					7	1	24															
23	2	2	4					7	1	24															
24	2	2	5					7	1	24															

Sex and Abdominal Status (SAS): 1 = Total Male; 2 = Unfed; 3 = Partly Fed; 4 = Fed; 5 = Gravid/semigravid; 6 = Total Female
Body Part (BP): 01 = Whole Adult; 13 = Eggs; 16 = Larvae; 17 = L1; 18 = L2; 19 = L3; 20 = L4; 21 = L1&L2; 22 = L3&L4; 23 = Pupae

Labelling details: First line: FT.SEN.FR
Second line: SLC.BP.ST.SID

Sample Sorting: Batch and/or Pool	Project Code (PC): Avecnet WP2	Experiment No. (EN): 1	SERIAL NO. (SEN): 0016
FORM TYPE (FT): SS3	ED Form Serial No. (SSEN): 1068	ED Form Row (SFR): 11	

Sorting/Obersvation Variables; Sample type variables

Form Row (FR)	Dead (DD) (1 = yes, 2 = no)	Taxon (TX)	Sex and Abdominal Status (SAS)	Mated (MT) (1 = yes, 2 = no)	Choice (CH)	Label (LA)	Finish Date (FD)	Finish Time (FT)	Body Form (BF)	Holding period (HP)	Other 5 (OTH5)	Other 6 (OTH6)	Other 7 (OTH7)	No. Observed (N)	Sample Lable Code (SLC)	No. of Individuals	Number of batches	Number in Batch 1 (SID = 01)	Number in Batch 2 (SID = 02)	Number in Batch 3 (SID = 03)	Number in Batch 4 (SID = 04)	Number in Batch 5 (SID = 05)	Number in Batch 6 (SID = 06)	Number in Batch 7 (SID = 07)	Number Discarded
MARK X FOR ALL VARIABLES WHICH MUST BE RECORDED	x	x	x																						
Define whether the attribute is categorical (CA) or continuous (CO)																									
																Sample type (ST):									
A	B	C	D	E	F	G	H	I	J	K	L	M	N	O	P	Q	R	S	T	U	V	W	X	Y	Z
																1		2	2	2	2	2	2	2	
01	1	1	2					7	1	0															
02	1	1	3					7	1	0				0											
03	1	1	4					7	1	0															
04	1	1	5					7	1	0															
05	1	2	2					7	1	0															
06	1	2	3					7	1	0				0											
07	1	2	4					7	1	0															
08	1	2	5					7	1	0															
09	1	1	2					7	1	24															
10	1	1	3					7	1	24															
11	1	1	4					7	1	24															
12	1	1	5					7	1	24															
13	1	2	2					7	1	24															
14	1	2	3					7	1	24				0											
15	1	2	4					7	1	24															
16	1	2	5					7	1	24															
17	2	1	2					7	1	24															
18	2	1	3					7	1	24				0											
19	2	1	4					7	1	24															
20	2	1	5					7	1	24															
21	2	2	2					7	1	24															
22	2	2	3					7	1	24				0											
23	2	2	4					7	1	24															
24	2	2	5					7	1	24															

Sex and Abdominal Status (SAS): 1 = Total Male; 2 = Unfed; 3 = Partly Fed; 4 = Fed; 5 = Gravid/semigravid; 6 = Total Female
Body Part (BP): 01 = Whole Adult; 13 = Eggs; 16 = Larvae; 17 = L1; 18 = L2; 19 = L3; 20 = L4; 21 = L1&L2; 22 = L3&L4; 23 = Pupae

Labelling details: First line: FT.SEN.FR
Second line: SLC.BP.ST.SID

Sample Sorting: Batch and/or Pool	Project Code (PC): Avecnet WP2	Experiment No. (EN): 1	SERIAL NO. (SEN): 0017
FORM TYPE (FT): SS3	ED Form Serial No. (SSEN): 1068	ED Form Row (SFR): 12	

Sorting/Obersvation Variables; Sample type variables

MARK X FOR ALL VARIABLES WHICH MUST BE RECORDED

Define whether the attribute is categorical (CA) or continuous (CO)

Form Row (FR)	Dead (DD) (1 = yes, 2 = no)	Taxon (TX)	Sex and Abdominal Status (SAS)	Mated (MT) (1 = yes, 2 = no)	Choice (CH)	Label (LA)	Finish Date (FD)	Finish Time (FT)	Body Form (BF)	Holding period (HP)	Other 5 (OTH5)	Other 6 (OTH6)	Other 7 (OTH7)	No. Observed (N)	Sample Lable Code (SLC)	No. of Individuals	Number of batches	Number in Batch 1 (SID = 01)	Number in Batch 2 (SID = 02)	Number in Batch 3 (SID = 03)	Number in Batch 4 (SID = 04)	Number in Batch 5 (SID = 05)	Number in Batch 6 (SID = 06)	Number in Batch 7 (SID = 07)	Number Discarded
MARK X	x	x	x																						
CA/CO																									
A	B	C	D	E	F	G	H	I	J	K	L	M	N	O	P	Q	R	S	T	U	V	W	X	Y	Z
Sample type (ST):																1		2	2	2	2	2	2	2	
01	1	1	2					7	1	0															
02	1	1	3					7	1	0				0											
03	1	1	4					7	1	0															
04	1	1	5					7	1	0															
05	1	2	2					7	1	0															
06	1	2	3					7	1	0				0											
07	1	2	4					7	1	0															
08	1	2	5					7	1	0															
09	1	1	2					7	1	24															
10	1	1	3					7	1	24															
11	1	1	4					7	1	24															
12	1	1	5					7	1	24															
13	1	2	2					7	1	24															
14	1	2	3					7	1	24				0											
15	1	2	4					7	1	24															
16	1	2	5					7	1	24															
17	2	1	2					7	1	24															
18	2	1	3					7	1	24															
19	2	1	4					7	1	24															
20	2	1	5					7	1	24															
21	2	2	2					7	1	24				0											
22	2	2	3					7	1	24															
23	2	2	4					7	1	24															
24	2	2	5					7	1	24															

Sex and Abdominal Status (SAS): 1 = Total Male; 2 = Unfed; 3 = Partly Fed; 4 = Fed; 5 = Gravid/semigravid; 6 = Total Female
Body Part (BP): 01 = Whole Adult; 13 = Eggs; 16 = Larvae; 17 = L1; 18 = L2; 19 = L3; 20 = L4; 21 = L1&L2; 22 = L3&L4; 23 = Pupae

Labelling details: First line: FT.SEN.FR
Second line: SLC.BP.ST.SID
